# A phase 1 clinical trial of the repurposable acetyllysine mimetic, *n*-methyl-2-pyrrolidone (NMP), in relapsed or refractory multiple myeloma

**DOI:** 10.1186/s13148-023-01427-7

**Published:** 2023-01-28

**Authors:** Jake Shortt, Peter Galettis, Chan Y. Cheah, Joanne Davis, Mandy Ludford-Menting, Emma K. Link, Jennifer H. Martin, Rachel Koldej, David Ritchie

**Affiliations:** 1 Blood Cancer Therapeutics Laboratory, Department of Medicine, School of Clinical Sciences at Monash Health, Faculty of Medicine, Nursing and Health Sciences, Monash University, Clayton, VIC Australia; 2 Monash Haematology, Monash Health, Clayton, VIC Australia; 3 Sir Peter MacCallum Department of Oncology, University of Melbourne, Parkville, VIC Australia; 4 Centre for Drug Repurposing and Medicines Research, University of Newcastle, Callaghan, NSW Australia; 5 Hunter Medical Research Institute, Kookaburra Circuit, New Lambton Heights, NSW Australia; 6 Department of Haematology, Sir Charles Gairdner Hospital, Perth, WA Australia; 7 Division of Internal Medicine, Medical School, University of Western Australia, Perth, WA Australia; 8 ACRF Translational Research Laboratory, Royal Melbourne Hospital, Melbourne, VIC Australia; 9 Department of Medicine, University of Melbourne, Melbourne, VIC Australia; 10 Centre for Biostatistics and Clinical Trials, Peter MacCallum Cancer Centre, Melbourne, VIC Australia; 11 Clinical Haematology, Peter MacCallum Cancer Centre and Royal Melbourne Hospital, Melbourne, VIC Australia

**Keywords:** *N*-methyl-2-pyrrolidone, Multiple myeloma, Bromodomain, Immunomodulation

## Abstract

**Background:**

*N*-methyl-2-pyrrolidone (NMP) is an epigenetically active chemical fragment and organic solvent with numerous applications including use as a drug-delivery vehicle. Previously considered biologically inert, NMP demonstrates immunomodulatory and anti-myeloma properties that are partly explained by acetyllysine mimetic properties and non-specific bromodomain inhibition. We therefore evaluated orally administered NMP in a phase 1 dose-escalation trial to establish its maximum tolerated dose (MTD) in patients with relapsed/refractory multiple myeloma (RR–MM). Secondary endpoints were safety, pharmacokinetics (PK), overall response rate and immunological biomarkers of activity.

**Results:**

Thirteen patients received NMP at starting doses between 50 and 400 mg daily. Intra-patient dose escalation occurred in five patients, with one attaining the ceiling protocolised dose of 1 g daily. Median number of monthly cycles commenced was three (range 1–20). Grade 3–4 adverse events (AEs) were reported in seven (54%; 95% CI 25–81%) patients. Most common AEs (> 30% of patients) of any grade were nausea and musculoskeletal pain. The only dose limiting toxicity (DLT) was diarrhoea in a patient receiving 200 mg NMP (overall DLT rate 8%; 95% CI 0–36%). Hence, the MTD was not defined. Median progression-free and overall survival were 57 (range 29–539) days and 33 (95% CI 9.7– > 44) months, respectively. The best response of stable disease (SD) was achieved in nine patients (69%; 95% CI 39–91%). PK analysis demonstrated proportional dose–concentrations up to 400 mg daily, with a more linear relationship above 500 mg. Maximum plasma concentrations (Cmax) of 16.7 mg/L at the 800 mg dose were below those predicted to inhibit BET-bromodomains. Peripheral blood immune-profiling demonstrated maintenance of natural killer (NK) cells, and a gene expression signature suggestive of enhanced T, B and NK cell functions; a subject with prolonged exposure manifested sustained recovery of B and NK cells at 12 months.

**Conclusions:**

NMP demonstrated potential disease stabilising and immunomodulatory activity at sub-BET inhibitory plasma concentrations and was well tolerated in RR–MM; an MTD was not determined up to a maximum dose of 1 g daily. Further dose-finding studies are required to optimise NMP dosing strategies for therapeutic intervention.

**Supplementary Information:**

The online version contains supplementary material available at 10.1186/s13148-023-01427-7.

## Background

*N*-methyl-2-pyrrolidone (NMP) is a fragment-sized organic solvent that is widely utilised for industrial applications. It is also used as a solubilising excipient for oral and transdermal routes of drug administration [1–3]. Although previously considered biologically inert, emerging data indicate NMP has pleiotropic biological activities including anti-inflammatory [4, 5], immunostimulatory [6, 7], bone protective/regenerative [8–10] and anti-atherogenic effects [11]. Mechanistically, NMP functions as an acetyllysine mimetic with low affinity but high ligand efficiency for bromodomains, including those of the BET-family [5, 12–14]. We have leveraged this observation to elaborate potent NMP-based BET bromodomain inhibitors [15, 16]. At relatively high concentrations (~ 1–10 mM), NMP phenocopies many effects of potent targeted BET-inhibitors, including suppression of lipopolysaccharide (LPS)-induced TNFα-secretion [4, 5] and downregulation of *cMYC* oncogene expression [5]. This observation likely explains the in vivo activity of NMP in a *cMYC-*driven mouse multiple myeloma model [5], systemic anti-inflammatory properties [5, 11], anti-atherosclerotic [11] and bone protective/regenerative [8, 9] activities. However, NMP also demonstrates immunomodulatory effects that are evident at sub-BET inhibitory concentrations of uncertain mechanism [5, 7].

Drug repurposing is an attractive strategy for accelerated research translation, potentially reducing development costs and timelines [17]. The safety and toxicity of NMP has previously been reported in the context of occupational exposures [1–3, 18, 19] and intentional overdose [20]. Healthy human volunteers have received up to 100 mg orally [3] and 300 mg transdermally [2] as a single dose with rapid absorption and no acute adverse effects. Examples of human pharmaceutical exposures include Eligard® (leuprorelin acetate depot) which contains up to 258.5 mg of NMP per dose [21]. We are unaware of any prior studies evaluating the safety or potential therapeutic activity of NMP in a repeated or dose-escalation format.

Multiple myeloma is an incurable plasma cell malignancy that is sensitive to both epigenetically targeted [22] and immunomodulatory therapies [23]. It is also hallmarked by significant skeletal complications, including bone lysis and osteoporosis [24]. Having serendipitously detected the activity of NMP in a mouse model of multiple myeloma [5], we sought to evaluate the potential safety and efficacy of NMP as a repurposable drug in this disease setting.

## Results

### Safety and tolerability

Thirteen patients commenced NMP between 2015 and 2020 when the trial was closed due to slow recruitment. Baseline patient demographics are summarised in Table 1. Subjects had received a median of four prior lines of therapy (range 2–9) with six (46%) being refractory to their most recent treatment. Three patients (23%) were treated in the ‘accelerated phase’ of the protocol, starting at doses between 50 and 200 mg per day. Five subjects underwent subsequent intra-patient dose escalation in the absence of a DLT or objective myeloma response. A summary of AEs is provided in Table 2. Two patients underwent dose level reductions for grade 2 non-haematological toxicities (bone pain and dizziness). Of note, five patients (38%) reported grade 1–2 musculoskeletal pains following NMP exposure. The other most common AEs were nausea (54%), reduced neutrophil counts (31%), anaemia (23%), diarrhoea (23%), anorexia (23%), peripheral sensory neuropathy (23%), dizziness (23%) and fatigue (23%). One patient (starting dose 200 mg/day) experienced grade 3 diarrhoea during cycle one requiring dose interruption, constituting the only DLT event for the trial. This subject was rechallenged at the 200 mg dose level from cycle two and remained on protocol at this dose until disease progression at cycle four. There were no grade 5 AEs.

**Table 1:** Patient demographics

Characteristic	Patients (*n* = 13)
Age, median [range]	73 [54–85]
Gender, *n* (%)	
Male	8 (62)
Female	5 (38)
ECOG performance status, *n* (%)	
0	7 (54)
1	6 (46)
Prior lines of therapy, *n* (%)	
2	4 (31)
3	1 (8)
> 3	8 (62)
Median prior lines [range]	4 [2–9]
Response to prior therapy, *n* (%)	
Refractory	6 (46)
Relapsed	4 (31)
Intolerant	3 (23)
Laboratory parameters, median [range]	
Creatinine clearance (mL/min)	84 [41–176]
β2 microglobulin (mg/L)	4.7 [2.0–13.6]
Albumin (g/L)	35 [29–45]
Paraprotein (g/L)	15 [0–52]
Kappa FLC (mg/L)	154 [5.2–1764]
Lambda FLC (mg/L)	5.8 [0.4–314]

*FLC* free light chains

**Table 2:** Adverse events

Adverse event	Worst grade (*n* = 13)					
	1	2	3	4	Total	
*Overall*	1	5	6	1	13	100%
Nausea	1	6	0	0	7	54%
Musculoskeletal pain^†^	2	3	0	0	5	38%
Neutrophil count decreased	0	1	2	0	3	23%
Anaemia	0	1	2	0	3	23%
Diarrhoea	2	0	1	0	3	23%
Anorexia	0	3	0	0	3	23%
Peripheral sensory neuropathy	0	3	0	0	3	23%
Dizziness	2	1	0	0	3	23%
Fatigue	3	0	0	0	3	23%
Fever	0	0	1	1	2	15%
Renal dysfunction^‡^	0	0	2	0	2	15%
URTI	0	2	0	0	2	15%
Constipation	1	1	0	0	2	15%
Pruritus	2	0	0	0	2	15%
Dysgeusia	2	0	0	0	2	15%
Sore throat	2	0	0	0	2	15%

Grading (severity) of adverse events regardless of relationship to study treatment and occurring in more than 10% of subjects

*LFT* liver function tests, *URTI* upper respiratory tract infection

^†^Musculoskeletal pain includes muscle or bone pain unrelated to bone marrow biopsy procedure

^‡^Renal dysfunction includes increase in blood urea, creatinine, reduced estimated glomerular filtration rate and acute renal failure

### Efficacy

**Fig. 1:**
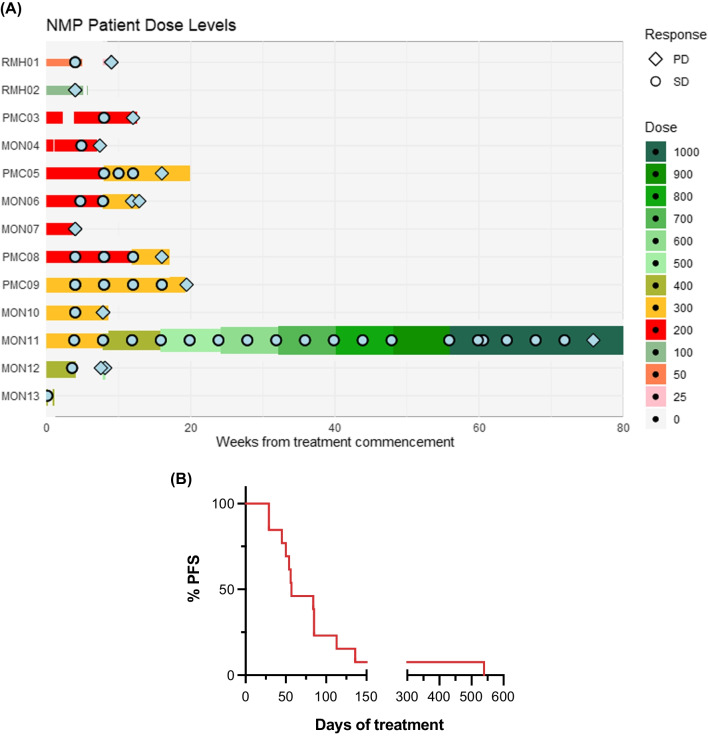
**A** Swimmer plots demonstrating progress of subjects treated for multiple myeloma with NMP from treatment initiation to discontinuation according to dose level. **B** Progression-free survival (PFS) of study participants from treatment initiation

**Fig. 2:**
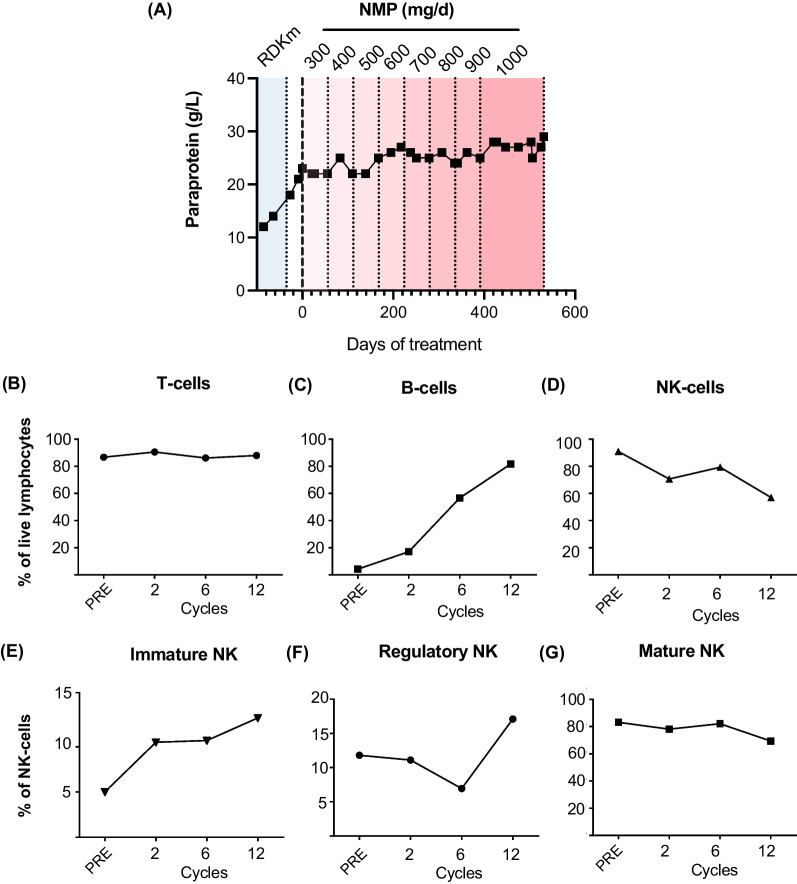
**A** Paraprotein results over time for patient achieving prolonged period of disease stabilisation on NMP treatment. NMP dose levels are indicated at the top of the graph. **B**–**G** Maintenance of immune cell subsets in the same patient from pre-treatment (PRE) through to cycle 12 on NMP therapy. **B** T cells (CD3^+^CD19^−^), **C** B cells (CD19^+^CD3^−^) and **D** NK cells (CD56^+^CD3^−^CD19^−^) are shown as percentage of live lymphocytes; **E** immature (CD16^−^CD56^hi^), **F** regulatory (CD16^−^CD56^+^), and **G** mature (CD16^+^CD56^+^) NK cell subsets are shown as percentage of live NK cells. RDKm, lenalidomide, dexamethasone, KappaMab

The disposition of patients according to best clinical response and duration on study is presented in Fig. 1A. All but one patient withdrew from the study due disease progression. No patient attained an International Myeloma Working Group (IMWG) defined objective disease response [25] (partial response or better, ORR 0%; 95% CI 0–25%). Nine patients were assessed as having stable disease (69%; 95% CI 39–91%). The median progression-free survival was 57 days (range 29–539 days; Fig. 1B). With a median follow-up of 43.5 months (95% CI 14.4–> 44.5 months), the median overall survival was 33.1 months (95% CI 9.7—> 44.5 months). Of note, one patient demonstrated a plateau in disease kinetics that was maintained until cycle 20. This patient was an 82 year old who had experienced disease progression while on lenalidomide, dexamethasone and KappaMab (MDX1097) [26] immediately prior to NMP treatment, having relapsed after initial treatment with bortezomib, cyclophosphamide and dexamethasone. As he did not experience any DLTs, he underwent serial dose level escalations up to the pre-specified ceiling NMP dose on the protocol of 1 g daily (Fig. 2A). Flow cytometry analysis of peripheral blood mononuclear cells (PBMCs) collected pre-treatment and after cycles two, six and 12 from this patient demonstrated maintenance of T and NK cells, and an increase in B cells (Fig. 2B–D). NK cell subset (immature, regulatory and mature) frequencies were also maintained throughout long-term NMP treatment (Fig. 2E–G and Additional file 1: Fig. S1–2).

### Pharmacokinetics

**Fig. 3:**
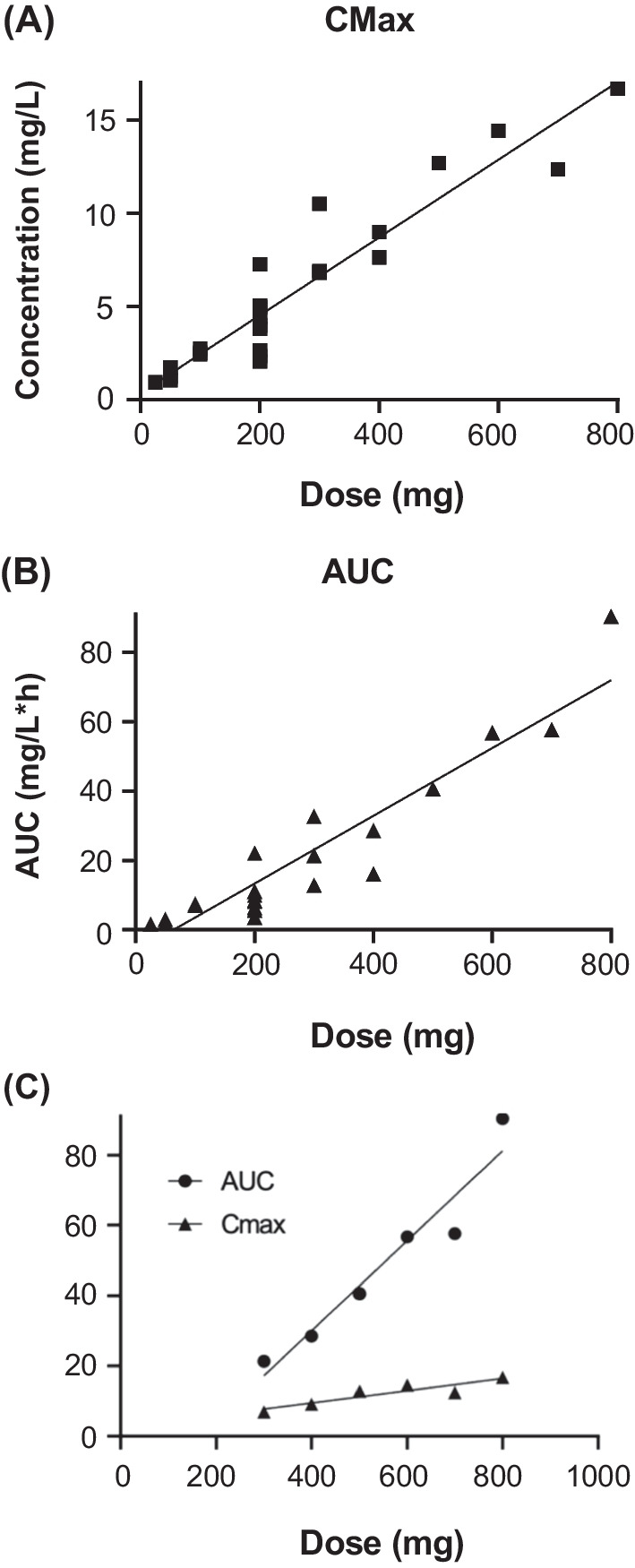
Pharmacokinetic analyses. **A** Relationship between dose of and Cmax for orally administered NMP. **B** Relationship between dose and AUC. **C** Relationship between dose and concentration for a single patient with multiple dose increments. Cmax, maximum plasma concentration; AUC, area under curve

Concentration time profiles were generated from 12 patients, with seven patients having one profile, one patient with two, one patient with three, one patient with four and two patients with six time profiles. Additional file 2: Table S1 provides the relationship of dose, concentration, area under the curve (AUC) and other relevant PK parameters obtained in this study. Where there was a single PK profile, there is one concentration and AUC; where there was more than one PK profile, concentration and AUC were averaged for each dose. Figure 3 illustrates the PK–Cmax versus dose for all patients (Fig. 3A), AUC versus dose for all patients (Fig. 3B) and Cmax and AUC versus dose for one patient who received multiple dose levels (Fig. 3C). The data indicate a dose–concentration curve that is relatively proportional until 800 mg. The patient who undertook serial dose escalations above 400 mg also showed an individual proportional dose–concentration curve between 400 and 800 mg. The mean half-life across all dose levels was 1.55 h, and the highest Cmax achieved was 16.66 mg/L (~ 168 µM) in the patient who was assessed at the 800 mg dose level. Pharmacokinetic studies were not performed above this dose level, although the same patient received NMP doses up to 1000 mg/day.

### Immunological biomarker studies

**Fig. 4:**
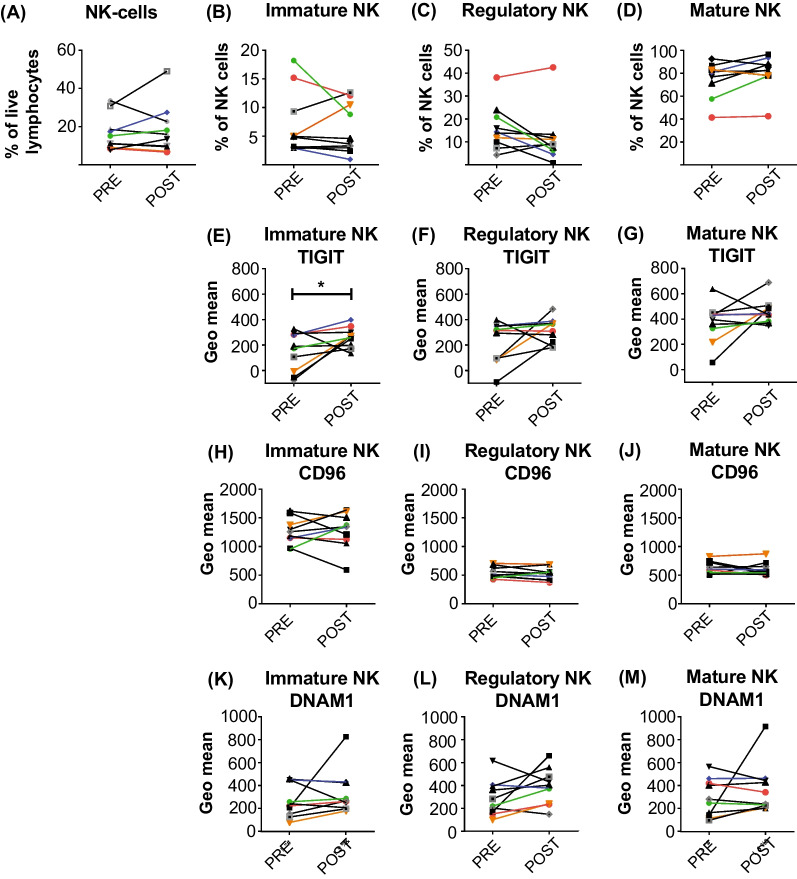
Maintenance of NK cell subsets in patients after NMP treatment. Flow cytometry analysis of PBMC from 10 patients collected pre-treatment (PRE) and after cycles 2–3 (POST) of NMP therapy. **A** NK cells (CD56^+^CD3^−^CD19^−^) are shown as a percentage of live lymphocytes, and **B** immature (CD16^−^CD56^hi^), **C** regulatory (CD16-CD56^+^) and **D** mature (CD16^+^CD56^+^) NK cell subsets are shown as a percentage of live NK cells*.* Expression of TIGIT (**E**–**G**), CD96 (**H**–**J**) and DNAM1 (**K**–**M**) was calculated using the geometric mean of the mature, regulatory and immature NK cell subsets. *p = 0.0371

**Fig. 5:**
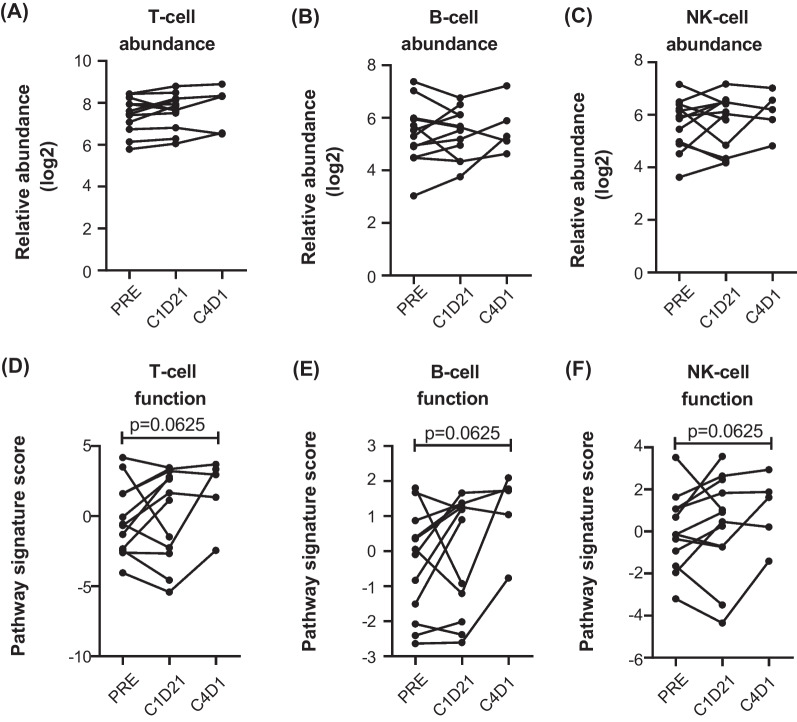
Gene expression immune profiling for abundance and function of immune cell subsets. The NanoString PanCancer Immune Profiling Panel V1 was used to analyse samples collected at pre-treatment (PRE), C1D21 and C4D1. The relative abundance of **A** T cells, **B** B cells or **C** NK cells, and the pathway signature scores indicating function of **D** T cells, **E** B cells and **F** NK cells are shown

Peripheral blood samples were collected for immune profiling by multicolour flow cytometry and NanoString® gene expression analysis with a focus on NK cells based on prior preclinical studies [5]. Flow cytometric analysis did not demonstrate an overall change in the proportion of T, B or NK cell proportions between baseline and the end of treatment cycle one (Additional file 1: Fig. S2, and Fig. 4A). NK subset analysis showed that the proportion of immature, regulatory and mature NK cells did not alter with NMP treatment (Fig. 4B–D). Expression of the NK receptors TIGIT, CD96 and DNAM1 on the NK cell subsets showed an increase in TIGIT on immature NK cells after 1–2 cycles of NMP treatment (Fig. 4E). It is likely that changes to NK receptor expression may be observed after longer periods of NMP treatment, as observed by the NanoString® abundance and function analysis (Fig. 5). Consistent with the flow cytometry data, NanoString® analysis also showed no changes in T, B or NK cell abundance (Fig. 5A–C); however, a trend towards increased T, B and NK cell function was noted following NMP treatment (Fig. 5D–F). Thus, commensurate with prior preclinical studies indicating that NMP may augment NK cell activity, we observed maintenance of NK cell populations and frequency, and a gene-expression signature suggesting enhanced T, B and NK cell functions post four cycles of NMP exposure.

## Discussion

This study investigated the potential to repurpose NMP as an immunologically active epigenetic fragment in the setting of RR–MM. NMP was generally well tolerated at starting doses up to 400 mg/day and intra-patient dose escalation culminated in a ceiling daily exposure of 1 g/day without definition of the MTD. The single observed DLT of diarrhoea, which was observed at the 200 mg/day dose level and did not recur with rechallenge at the same dose, may not have been NMP related. Moreover, many of the commonly reported low grade AEs (e.g. musculoskeletal pains, renal impairment and peripheral neuropathy) are frequent manifestations of underlying myeloma confounding causality assessments. An association between NMP exposure and bone pain was observed which may indicate a treatment-related event. Prior reports of augmented bone morphogenic protein signalling [8], inhibition of osteoclast differentiation [10], enhanced bone tissue regeneration [8] and anti-osteoporotic [9] effects in response to NMP may indicate that the bone pains observed in this study relate to modulation of bone metabolism. As bone lysis is a hallmark of myeloma, the potential bone protective effects of NMP warrant further investigation.

NMP disposition in humans has mainly been studied from a toxicology and toxicokinetic perspective, to minimise adverse effects from inhalation in industrial settings [27]. A human study demonstrated intact NMP (0.8%) with metabolites 5-hydroxy-*N*-methyl-2-pyrrolidone (5-HNMP—44%), *N*-methylsuccinimide (MSI—0.4%) and 2-hydroxy-*N*-methylsuccinimide (2-HMSI—20%) in urine after a single 100 mg oral dose. One-third of the oral NMP was not recovered as either the parent or these metabolites, with the lack of mass balance potentially accounted for other metabolites including 2-pyrrolidone and other succinimides [3]. Our data are consistent with this report, indicating that intact NMP is rapidly absorbed (Cmax attained in ~ 0.5 h) with a short half-life (~ 1.5 h). Peak plasma NMP concentrations were approximately tenfold–100-fold lower than those required to functionally inhibit BET-bromodomains and induce a BET-inhibitory signature in cell-based assays [5]. This is consistent with the absence of typical BET-inhibitor toxicities (e.g. thrombocytopenia) observed in the patient cohort. Based on NMP’s PK, we predict that single NMP doses more than 4.5 g would be required to attain BET-inhibitory peak plasma concentrations (e.g. > 1 mM) which may not be clinically feasible. We did not measure NMP metabolites in this study, but previous data indicate considerably longer elimination half-lives for MSI (8 h) and 2-HMSI (17 h) than NMP itself [3]. Thus, patient exposures to NMP metabolites are likely much greater than that of the parent molecule. As the primary metabolic pathway is initiated by 5-hydroxylation, the primary NMP metabolites (5-HNMP, MSI and 2-HMSI) are not predicted to mediate bromodomain inhibition.

Although no patient demonstrated an IMWG-defined objective treatment response, potential disease stabilising activity was noted with four patients remaining on study more than 100 days and one subject demonstrating disease control for up to 20 cycles. Correlation of PK data with preclinical studies indicates that any anti-myeloma activity is not readily explained by the direct effects of NMP-mediated BET-bromodomain inhibition on tumour cells. We therefore posited that disease control could be immune mediated, noting that NMP may modulate immune function at micromolar concentrations achieved in these patients [5, 7]. Commensurate with this hypothesis, we observed maintenance of NK populations, and a gene expression signature suggestive of post-NMP enhancement of T cell and NK cell function. In the patient with a more sustained NMP exposure, there was evidence of improved immunity with maintenance of NK cells and recovery of B cells at 12 months. The short period between of baseline and on-treatment samples likely reduced the ability to observe improvements immune function in most patients. Moreover, the rapid onset of disease progression in this heavily pre-treated cohort of patients further limited the opportunity for observation of immune changes. Nonetheless, the observation of early changes in gene expression indicates that patients with less aggressive myeloma may show beneficial improvement in markers of immunity with more protracted exposure to NMP. This activity remains mechanistically undetermined but could relate to promiscuous low-potency bromodomain interactions outside of the BET-family [14] or non-bromodomain containing targets.

## Conclusions

*N*-methyl-2-pyrrolidone can be safely administered to patients with RR–MM at starting doses up to 400 mg/day without recurring dose limiting toxicities. Potential disease stabilising activity correlated with biomarkers of preserved immune function and was observed at sub-BET inhibitory plasma concentrations. Future dose finding studies are required to determine the maximum tolerated NMP dose and mechanistic activity beyond BET-bromodomain inhibition.

## Methods

### Study design

This was a phase 1, open-label dose-escalation trial of orally administered NMP in patients with relapsed/refractory multiple myeloma (RR–MM). Eligible patients were ≥ 18 years old with Eastern Cooperative Oncology Group (ECOG) < 2 and histologically confirmed plasma cell myeloma defined by WHO 2008 criteria with measurable disease (defined by serum M-protein > 5 g/L, urine M-protein > 200 mg/24 h, involved serum free light chain > 100 mg/L or measurable soft tissue plasmacytoma) [28]. Patients were required to have relapsed following prior treatment and/or be refractory to or intolerant of both bortezomib and lenalidomide and must have undergone autologous stem cell transplant (unless deemed ineligible at investigator discretion). Exclusion criteria included: central nervous system involvement, active second malignancy within two years, uncontrolled medical conditions contraindicating an investigational agent due to inadequate organ function and concomitant exposure to anti-myeloma therapies.

The primary study endpoint was to determine the MTD of orally administered NMP in patients with RR–MM. Secondary endpoints included safety, PK analyses, IMWG defined response rate and exploratory immunological biomarkers. Adverse events were defined according to Common Terminology Criteria for Adverse Events (CTCAE version 4.03). Haematological DLTs were defined as grade 4 neutropenia unresponsive to G-CSF, grade 4 thrombocytopenia and febrile neutropenia. Non-haematological DLTs were grade 3 or higher and considered probably related to study drug and not attributable to disease progression and those resulting treatment delays greater than two weeks due to drug-related toxicity. Self-limited (< 24 h) gastrointestinal side effects (e.g. nausea, diarrhoea) responding to supportive measures were not classified as DLTs.

Pharmaceutical grade NMP stock solution (Ashland, Columbus, OH, USA) was diluted in ORA SWEET® SF (Medical Flavouring Systems, Vic, Australia) oral syrup vehicle and sterile water to a final concentration of 50 mg/mL and dispensed weekly in amber glass bottles for storage a room temperature. NMP was administered as a single morning dose on an empty stomach at least 30 min prior to food. Patients were dosed according to an ‘accelerated’ escalation schedule (one patient per dose level) in the absence of significant toxicity until a DLT was experienced and then in a 3 × 3 ‘standard’ dose-escalation phase. Intra-patient dose escalation at 100 mg increments was also permitted whereby the MTD had not been determined and the patient tolerated two consecutive cycles without DLT or an objective disease response. Treatment was administered daily for 28-day cycles until withdrawal from protocol due to toxicity or disease progression.

### Pharmacokinetic analysis

Blood samples were taken from patients before NMP and at one, two, four, eight and 24 h post dose. Plasma samples (20 μl) were prepared by adding five volumes of methanol containing deuterated internal standard. The samples were vortexed then centrifuged, and the supernatant was transferred to a vial and injected onto the LCMSMS. The LCMSMS system consisted of a Shimadzu UHPLC with a SCIEX 6500QTrap, a KinetexC18 (3 × 50 mm, 2.6 μm) column and using a gradient of 0.1% formic acid and acetonitrile. The initial conditions were 0.5 ml/min and 2% acetonitrile; this was held for 1 min then increased to 20% acetonitrile at 2 min then increased to 70% acetonitrile and held for 0.5 min. Equilibration time was 0.5 min. MRMs for NMP were 100 → 58, 100 → 69, 100 → 41 and for the internal standard d9NMP 109 → 62, 100 → 61, 100 → 46. PK analysis was performed using Pkanalix (version 2021R1. Antony, France: Lixoft SAS, 2021).

### Blood samples for immunological biomarkers

Peripheral blood was collected at study enrolment, and at monthly intervals after each cycle of treatment. PBMC was isolated using Ficoll-Paque Plus (GE Healthcare, Chicago, IL, USA) and cryopreserved until required for analysis. Ten patients had sufficient PBMC stored for analysis (screening or C1D1 (pre-treatment), and C2D1 or C3D1 (post)). Four patients had C4D1 samples collected, and one patient on long-term NMP treatment had C6D1, and C12D1 samples analysed.

### Flow cytometry

PBMC was stained using Zombie Live/dead fixable cell stain (Thermo Fisher Scientific, Waltham, MA, USA) prior to staining with specific antibodies. Antibodies used were CD3 BUV496 (BD Bioscience), CD19 PeCy7 (BD Bioscience), CD56 BB700 (BD Bioscience), CD16 BV650 (BioLegend), CD96 BV421 (BD Bioscience), TIGIT BUV395 (BD Bioscience), DNAM1 (CD226) BUV805 (BD Bioscience). PBMC was stained in Live/Dead cell stain for 15 min in PBS at RT, washed in FACS buffer (PBS + 2% FCS), and blocked in Human Fc block (BD Bioscience) for five minutes at RT. Cell staining was performed in FACS buffer for 30 min at 4 °C, followed by two washes in FACS buffer, and fixation in 2% paraformaldehyde (PFA, Electron Microscopy Sciences). Samples were acquired on a LSR Fortessa (BD Bioscience) flow cytometer and analysed using FlowJo software (BD Bioscience). Flow cytometry gating is shown in Additional file 1: Fig. S1. NK cells were defined as CD3-CD19-CD56 + viable single lymphocytes. NK cell subsets were defined as mature (CD16 + CD56mid), regulatory (CD16-CD56mid) and immature (CD16-CD56hi). Expression of DNAM1, TIGIT and CD96 was calculated using the geometric mean of the mature, regulatory and immature NK cell subsets.

### Gene expression analysis

NanoString gene expression profiling of PBMC was performed as previously described [29] using NanoString PanCancer Immune Profiling Panel V1 (NanoString Technologies, Seattle, WA, USA). Data were normalised and analysed using nCounter Advanced Analysis software (version 2.0.115; NanoString Technologies).

### Statistical analyses

The study was planned to recruit up to 20 patients, 15 in the dose finding cohort and five as a dose expansion. A pragmatic sample of 15 patients would yield a 95% confidence interval between 4 and 48% for an actual grade 3–4 event rate of 20% which was considered suitable precision for a phase 1 pilot study. Time to progression was calculated from the first day of treatment to progression using the Kaplan–Meier method. Flow cytometry and gene expression data were analysed using GraphPad Prism (version 6.0; GraphPad Software, San Diego, CA). Data were analysed using Wilcoxon’s matched pairs signed rank test compared with baseline samples (*p < 0.05).

## Supplementary Information

**Additional file 1. Fig S1:** Flow cytometry gating strategy to identify NK cell subsets. Single cells were stratified into lymphocyte and monocyte populations based on SSC-A vs FSC-A, and gated on live lymphocytes (Zombie negative). NK cells (CD56+) were gated on non T and B cells (CD3-CD19-). NK cell subsets were defined as mature (CD16+CD56+), regulatory (CD16-CD56+), and immature (CD16-CD56hi). **Fig S2:**Maintenance of T and B cells in patients on NMP treatment. Flow cytometry analysis of peripheral blood mononuclear cells (PBMC) from 10 patients collected pre-treatment and after cycles 2-3 (post) on NMP therapy. T cells (CD3+CD19-) (A), and B cells (CD19+CD3-) (B) are shown as a percentage of live lymphocytes.

**Additional file 2. Table S1.** Relationship between dose and PK parameters for the study. Tmax, time to maximum plasma concentration; Cmax, maximum plasma concentration; AUC, area under curve; CL/F, oral clearance; V, volume; F, bioavailability; SD, standard deviation; CV, coefficient of variation. Note: for multiple PK samples on the same dose, parameters were averaged.

## Data Availability

De-identified patient data will be shared upon request within three years of publication subject to a data sharing agreement and an ethically approved research proposal.

## Abbreviations

AUC: Area under curve
Cmax: Maximum plasma concentration
DNAM-1: DNAX accessory molecule-1
DLT: Dose limiting toxicity
IMWG: International Myeloma Working Group
LCMSMS: Liquid chromatography tandem mass spectrometry
MTD: Maximum tolerated dose
NMP: *N*-methyl-2-pyrrolidone
NK: Natural killer
PBMC: Peripheral blood mononuclear cell
PK: Pharmacokinetic
TIGIT: T cell immunoreceptor with Ig and ITIM domains
