# Supplementary material for: A phase 1 clinical trial of the repurposable acetyllysine mimetic, n-methyl-2-pyrrolidone (NMP), in relapsed or refractory multiple myeloma
Source: Clin Epigenetics. 2023 Jan 28;15:15. doi: 10.1186/s13148-023-01427-7 (PMC9884426; doi:10.1186/s13148-023-01427-7)
Supplement: Supplementary file 1 — Additional file 1. Fig S1: Flow cytometry gating strategy to identify NK cell subsets. Single cells were stratified into lymphocyte and monocyte populations based on SSC-A vs FSC-A, and gated on live lymphocytes (Zombie negative). NK cells (CD56+) were gated on non T and B cells (CD3-CD19-). NK cell subsets were defined as mature (CD16+CD56+), regulatory (CD16-CD56+), and immature (CD16-CD56hi). Fig S2:Maintenance of T and B cells in patients on NMP treatment. Flow cytometry analysis of peripheral blood mononuclear cells (PBMC) from 10 patients collected pre-treatment and after cycles 2-3 (post) on NMP therapy. T cells (CD3+CD19-) (A), and B cells (CD19+CD3-) (B) are shown as a percentage of live lymphocytes. [file 13148_2023_1427_MOESM1_ESM.pptx]

## Slide 1
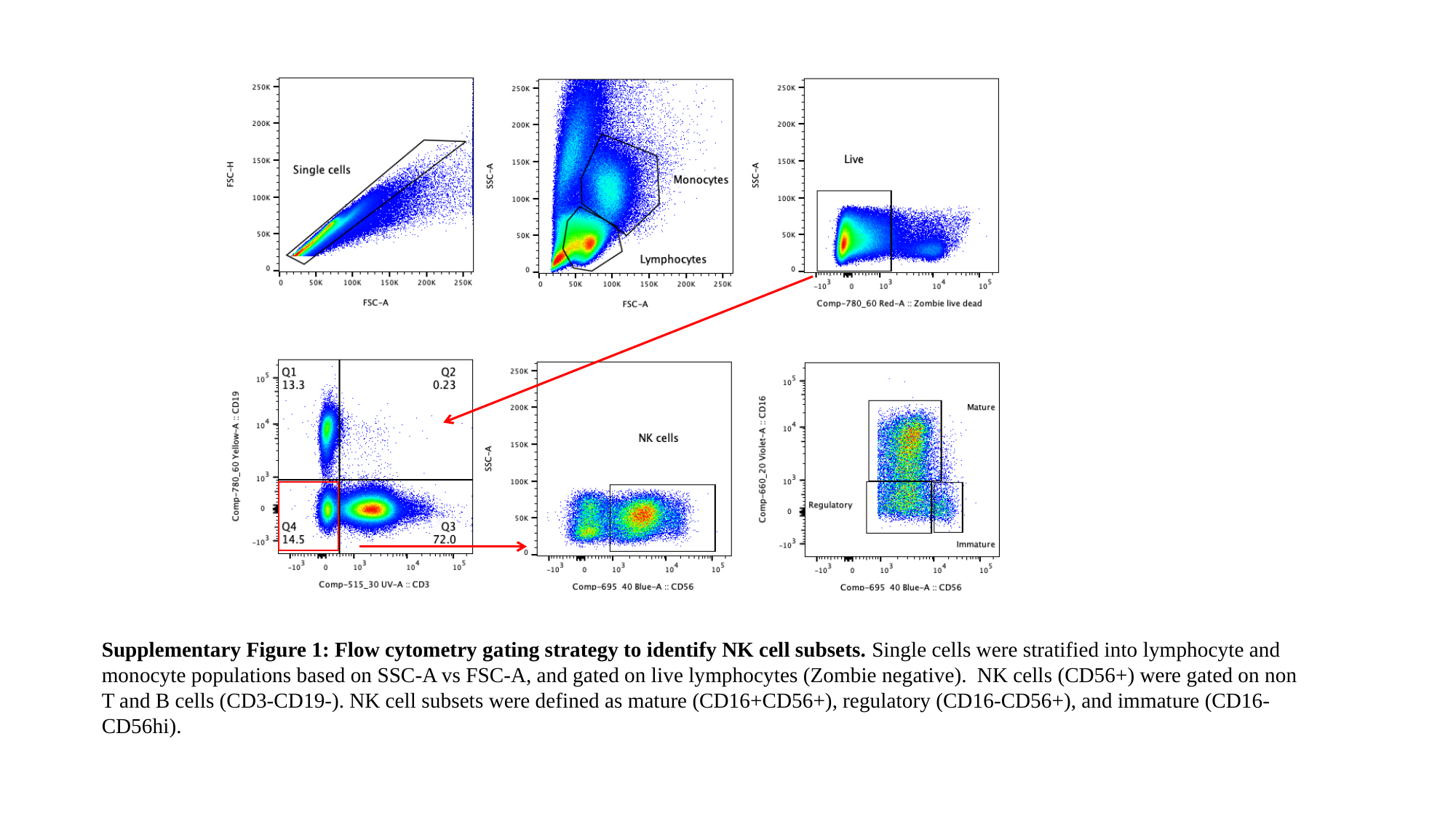

Supplementary Figure 1: Flow cytometry gating strategy to identify NK cell subsets. Single cells were stratified into lymphocyte and monocyte populations based on SSC-A vs FSC-A, and gated on live lymphocytes (Zombie negative). NK cells (CD56+) were gated on non T and B cells (CD3-CD19-). NK cell subsets were defined as mature (CD16+CD56+), regulatory (CD16-CD56+), and immature (CD16-CD56hi).

## Slide 2
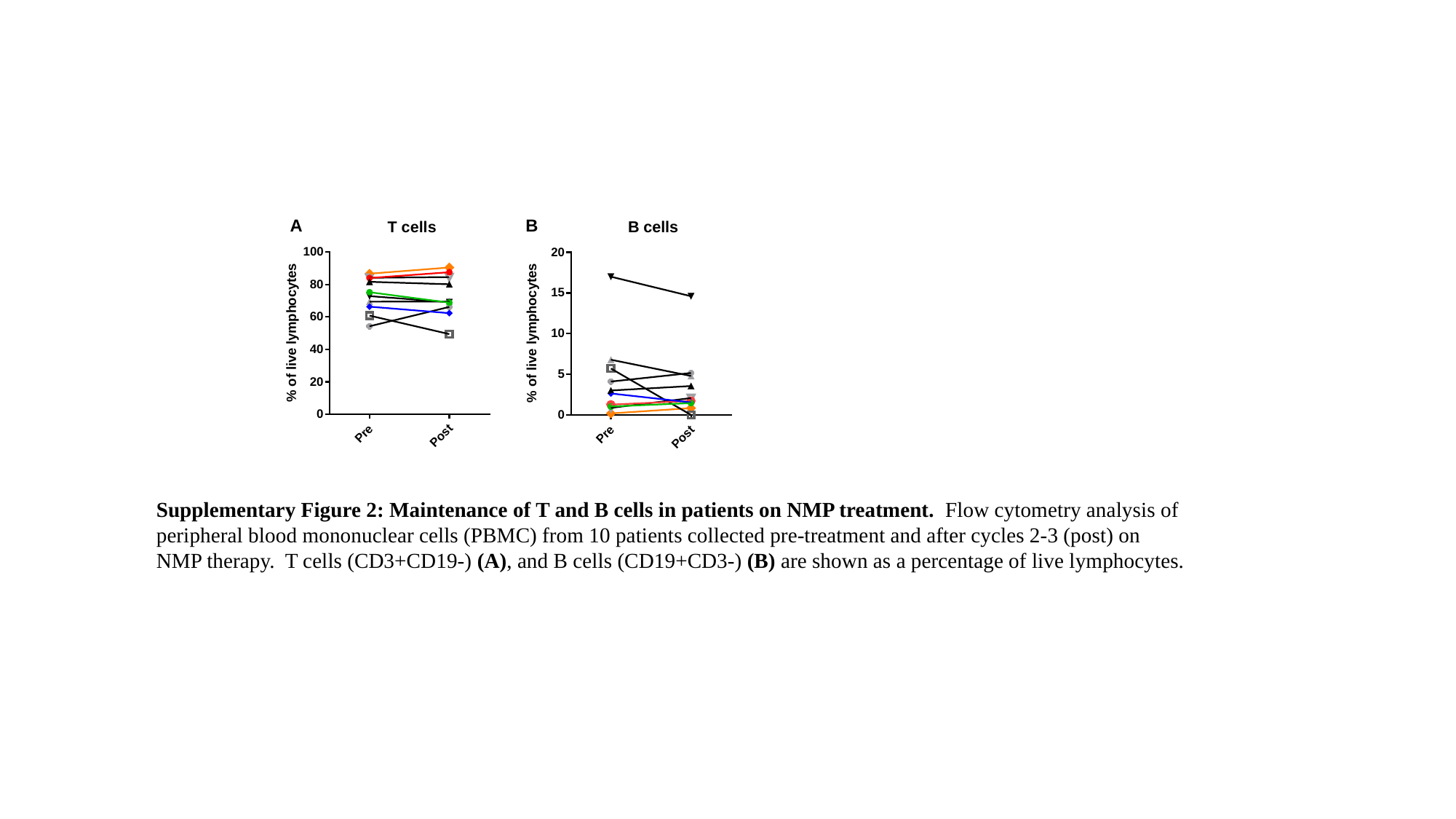

Supplementary Figure 2: Maintenance of T and B cells in patients on NMP treatment. Flow cytometry analysis of peripheral blood mononuclear cells (PBMC) from 10 patients collected pre-treatment and after cycles 2-3 (post) on NMP therapy. T cells (CD3+CD19-) (A), and B cells (CD19+CD3-) (B) are shown as a percentage of live lymphocytes.
