# Supplementary material for: A phase 1 clinical trial of the repurposable acetyllysine mimetic, n-methyl-2-pyrrolidone (NMP), in relapsed or refractory multiple myeloma
Source: Clin Epigenetics. 2023 Jan 28;15:15. doi: 10.1186/s13148-023-01427-7 (PMC9884426; doi:10.1186/s13148-023-01427-7)
Supplement: Supplementary file 2 — Additional file 2. Table S1. Relationship between dose and PK parameters for the study. Tmax, time to maximum plasma concentration; Cmax, maximum plasma concentration; AUC, area under curve; CL/F, oral clearance; V, volume; F, bioavailability; SD, standard deviation; CV, coefficient of variation. Note: for multiple PK samples on the same dose, parameters were averaged. [file 13148_2023_1427_MOESM2_ESM.docx]

| **Patient ID** | **Occasion** | **Dose (mg)** | **Tmax (h)** | **Cmax (mg⋅L⁻¹)** | **AUC (h⋅mg⋅L⁻¹)** | **CL/F (L⋅h⁻¹)** | **V/F**  **(L)** | **Half-life**  **(h)** |
| --- | --- | --- | --- | --- | --- | --- | --- | --- |
| RMH-01 | 6 | 25 | 0.5 | 0.93 | 1.5 | 16.65 | 20.83 | 0.87 |
|  |  |  |  |  |  |  |  |  |
| RMH-01 | 1 | 50 | 0.5 | 1.72 | 2.83 | 17.68 | 30.85 | 1.21 |
| RMH-01 | 2 | 50 | 0.5 | 1.24 | 1.86 | 26.86 | 38.66 | 0.99 |
| RMH-01 | 3 | 50 | 0.5 | 1.25 | 1.88 | 26.66 | 41.02 | 1.07 |
| RMH-01 | 4 | 50 | 0.5 | 1.04 | 2.09 | 23.93 | 35.88 | 1.04 |
| RMH-01 | 5 | 50 | 0.5 | 1.06 | 2.18 | 22.97 | 36.67 | 1.11 |
|  | **Average** |  | 0.50 | 1.26 | 2.17 | 23.62 | 36.62 | 1.08 |
|  | **SD** |  | 0.00 | 0.27 | 0.39 | 3.73 | 3.79 | 0.08 |
|  | **CV** |  | 0 | 22 | 18 | 16 | 10 | 8 |
|  | **N** |  | 5 | 5 | 5 | 5 | 5 | 5 |
|  |  |  |  |  |  |  |  |  |
| RMH-02 | 1 | 100 | 0.5 | 2.73 | 6.97 | 14.35 | 34.92 | 1.69 |
| RMH-02 | 2 | 100 | 0.5 | 2.51 | 7.25 | 13.79 | 41.77 | 2.1 |
| RMH-02 | 3 | 100 | 0.5 | 2.52 | 6.95 | 14.4 | 37.5 | 1.81 |
| RMH-02 | 4 | 100 | 0.5 | 2.43 | 7.61 | 13.13 | 34.02 | 1.8 |
| RMH-02 | 5 | 100 | 0.5 | 2.65 | 6.88 | 14.53 | 36.67 | 1.75 |
|  | **Average** |  | 0.50 | 2.57 | 7.13 | 14.04 | 36.98 | 1.83 |
|  | **SD** |  | 0.00 | 0.12 | 0.30 | 0.58 | 3.01 | 0.16 |
|  | **CV** |  | 0 | 5 | 4 | 4 | 8 | 9 |
|  | **N** |  | 5 | 5 | 5 | 5 | 5 | 5 |
|  |  |  |  |  |  |  |  |  |
| PMC-03 | 1 | 200 | 0.5 | 2.33 | 6.29 | 31.82 | 64.35 | 1.4 |
| PMC-03 | 2 | 200 | 0.5 | 3.98 | 8.33 | 24.02 | 55.72 | 1.61 |
| PMC-03 | 3 | 200 | 0.5 | 4.43 | 7.98 | 25.05 | 43.19 | 1.2 |
| MON-04 | 1 | 200 | 0.5 | 5.05 | 9.83 | 20.34 | 37.65 | 1.28 |
| PMC-05 | 1 | 200 | 1 | 2.05 | 5.49 |  |  |  |
| MON-06 | 1 | 200 | 0.5 | 4.95 | 10.99 | 18.2 | 36.79 | 1.4 |
| MON-06 | 2 | 200 | 0.5 | 7.26 | 21.99 | 9.1 | 26.02 | 1.98 |
| MON-07 | 1 | 200 | 0.5 | 2.64 | 3.46 | 57.76 | 87.19 | 1.05 |
| PMC-08 | 1 | 200 | 0.5 | 3.79 | 11.21 | 17.85 | 47.25 | 1.83 |
|  | **Average** |  | 0.56 | 4.05 | 9.51 | 25.52 | 49.77 | 1.47 |
|  | **SD** |  | 0.17 | 1.63 | 5.34 | 14.59 | 19.19 | 0.32 |
|  | **CV** |  | 30 | 40 | 56 | 57 | 39 | 22 |
|  | **N** |  | 9 | 9 | 9 | 8 | 8 | 8 |
|  |  |  |  |  |  |  |  |  |
| PMC-09 | 1 | 300 | 0.5 | 10.49 | 32.55 | 9.22 | 22.09 | 1.66 |
| MON-10 | 1 | 300 | 0.5 | 6.89 | 12.73 | 23.57 | 38.59 | 1.13 |
| MON-11 | 1 | 300 | 1 | 6.81 | 21.3 | 14.08 | 41.1 | 2.02 |
|  | **Average** |  | 0.67 | 8.06 | 22.19 | 15.62 | 33.93 | 1.60 |
|  | **SD** |  | 0.29 | 2.10 | 9.94 | 7.30 | 10.33 | 0.45 |
|  | **CV** |  | 43 | 26 | 45 | 47 | 30 | 28 |
|  | **N** |  | 3 | 3 | 3 | 3 | 3 | 3 |
|  |  |  |  |  |  |  |  |  |
| MON-11 | 2 | 400 | 0.5 | 8.99 | 28.51 | 14.03 | 35.95 | 1.78 |
| MON-12 | 1 | 400 | 0.5 | 7.63 | 15.99 | 25.02 | 48.93 | 1.36 |
|  | **Average** |  | 0.50 | 8.31 | 22.25 | 19.53 | 42.44 | 1.57 |
|  | **SD** |  | 0.00 | 0.96 | 8.85 | 7.77 | 9.18 | 0.30 |
|  | **CV** |  | 0 | 12 | 40 | 40 | 22 | 19 |
|  | **N** |  | 2 | 2 | 2 | 2 | 2 | 2 |
|  |  |  |  |  |  |  |  |  |
|  |  |  |  |  |  |  |  |  |
|  |  |  |  |  |  |  |  |  |
| **Subject ID** | **Occasion** | **Dose (mg)** | **Tmax (h)** | **Cmax (mg⋅L⁻¹)** | **AUC (h⋅mg⋅L⁻¹)** | **CL/F (L⋅h⁻¹)** | **V/F (L)** | **Half-life (h)** |
| MON-11 | 3 | 500 | 0.5 | 12.69 | 40.57 | 12.33 | 35.2 | 1.98 |
|  |  |  |  |  |  |  |  |  |
| MON-11 | 4 | 600 | 0.5 | 14.41 | 56.67 | 10.59 | 33.11 | 2.17 |
|  |  |  |  |  |  |  |  |  |
| MON-11 | 5 | 700 | 0.5 | 12.35 | 57.58 | 12.16 | 41.26 | 2.35 |
|  |  |  |  |  |  |  |  |  |
| MON-11 | 6 | 800 | 1 | 16.66 | 90.3 | 8.86 | 22.43 | 1.75 |
|  |  |  |  |  |  |  |  |  |
| **Overall** | **Average** |  | 0.55 | 5.29 | 16.89 | 19.25 | 39.49 | 1.55 |
|  | **SD** |  | 0.15 | 4.39 | 20.81 | 9.79 | 13.19 | 0.41 |
|  | **CV** |  | 28 | 83 | 123 | 51 | 33 | 27 |
|  | **N** |  | 29 | 29 | 29 | 28 | 28 | 28 |

**Supplementary Table 1.** Relationship between dose and PK parameters for the study. Tmax, time to maximum plasma concentration; Cmax, maximum plasma concentration; AUC, area under curve; CL/F, oral clearance; V, volume; F, bioavailability; SD, standard deviation; CV, coefficient of variation. Note: for multiple PK samples on the same dose, parameters were averaged.
